# Supplementary material for: Waveforms of molecular oscillations reveal circadian timekeeping mechanisms
Source: Commun Biol. 2018 Nov 26;1:207. doi: 10.1038/s42003-018-0217-1 (PMC6255756; doi:10.1038/s42003-018-0217-1)
Supplement: Supplementary file 1 — Supplementary Information [file 42003_2018_217_MOESM1_ESM.pdf]

Supplementary Information of "Waveforms of molecular oscillations reveal circadian timekeeping mechanisms"

Supplementary Methods

Analysis of PRR7 data. In the case of the *PRR7* gene in the *Arabidopsis* circadian system, we obtained the experimental data of the mRNA and protein profiles from Fig. 5(d) of Flis et al.[1] and Fig. 5(a) of Nakamichi et al.[2], respectively. Both datasets have 2-hour sampling intervals under 12L:12D cycles. These mRNA and protein levels were normalized by the peak levels of their splines, and adopted for $g_{\mathrm{m}}(t)$ and $x(t)$ in Eq. (18), respectively. We obtained the experimental protein degradation rates by fitting exponential functions to the protein abundance data in Fig. 7(b) of Farre et al.[3] from seedlings treated with CHX at ZT4 and then kept in the light, and from seedlings treated with CHX at ZT12 and transferred to darkness. They were adopted for $r(t)$ at $t = 4$ h and 12 h ($r(t = 4\text{ h}) \approx 0.09\text{ h}^{-1}$ and $r(t = 12\text{ h}) \approx 0.34\text{ h}^{-1}$). In addition, we used our own experimental data that lead to $r(t = 18\text{ h}) \approx 0.45\text{ h}^{-1}$, as presented in Supplementary Fig. 1 and in the upper panel of Supplementary Fig. 2. In this experiment, we measured the protein levels after CHX treatment at ZT17, and considering a lag time for the full effect of CHX, we fitted an exponential function to the data from ZT18 (Supplementary Fig. 2). This $r(t = 18\text{ h})$, as well as $r(t = 4\text{ h})$ and $r(t = 12\text{ h})$, satisfies Eq. (3) (Fig. 2b). Alternatively, an exponential fit from ZT17 leads to $r(t = 17\text{ h}) \approx 0.31\text{ h}^{-1}$ (Supplementary Fig. 2, lower panel), still supporting Eq. (3). If we fit an exponential function to the entire data from all biological repeats at ZT18, $r(t = 18\text{ h}) \approx 0.45 \pm 0.11\text{ h}^{-1}$ (avg. $\pm$ s.d.). This standard deviation of $r(t = 18\text{ h})$ does not much

change our main result presented below, because it leads to $c/c_{\mathrm{g}} \approx 0.27 \sim 0.32$.

As described in Methods for constant translation rate $k(t) \approx k$, once k is estimated from Eq. (19) with the experimental protein profile $x(t)$, mRNA profile $g_{\mathrm{m}}(t)$, and degradation rate $r(t)$s at $t = 4$ h, 12 h, and 18 h, one can infer the entire $r(t)$ over the course of a day using Eq. (20) (for the contrary case, i.e., time-varying $k(t)$, refer to the analysis in Methods). Because experimental protein and mRNA levels have 2-hour sampling intervals, we infer $r(t)$ every 2 hours, except for $t = 4$ h, 12 h, and 18 h for which we use experimentally-known $r(t)$ values. The overall $r(t)$ profile exhibits two peaks at 20 h $\leq t \leq$ 22 h and at 2 h $\leq t \leq$ 10 h. The former peak is a natural consequence of large $R(t)$ around that time (red solid line in Supplementary Fig. 3a), while the latter may be an artifact from unconsidered biological factors. To reduce the effect of such possible artifact, we replace every $r(t) > \max_{20\,\mathrm{h} \leq t \leq 22\,\mathrm{h}} r(t)$ by $\max_{20\,\mathrm{h} \leq t \leq 22\,\mathrm{h}} r(t)$, because $\max_{20\,\mathrm{h} \leq t \leq 22\,\mathrm{h}} r(t) \approx 1.02$ h^{-1} and the real degradation rate is unlikely to be larger than 1.02 h^{-1}. We also replace every $r(t) < \min\{r(t = 4\,\mathrm{h}), r(t = 12\,\mathrm{h}), r(t = 18\,\mathrm{h})\}$ by $\min\{r(t = 4\,\mathrm{h}), r(t = 12\,\mathrm{h}), r(t = 18\,\mathrm{h})\}$, and therefore the lower bound of $r(t)$ is set to the minimum value of experimental $r(t)$ values. In such a way, the difference between c and c_{g} is reduced (Eqs. (2) and (5)), leading to a conservative estimate of that difference. The resulting $r(t)$ is presented in Supplementary Fig. 3a. Because $r(t)$ at 2 h $\leq t \leq$ 10 h is improbably deviated from the overall trend of experimental $r(t)$ values, we correct this part by linear interpolation and extrapolation of the experimental $r(t = 4\,\mathrm{h})$ and $r(t = 12\,\mathrm{h})$ values, as shown in Fig. 2f. Consequently, $c \approx 0.30 c_{\mathrm{g}}$ with $r(t)$ in Fig. 2f and $c \approx 0.67 c_{\mathrm{g}}$ with $r(t)$ in Supplementary Fig. 3a. In other words, whether correcting $r(t)$ at 2 h $\leq t \leq$ 10 h or not, the actual cost of PRR7 waveform maintenance would be

at most one- to two-thirds of the assumed cost in the case of a constant degradation rate.

Thus far, we have adopted the experimental protein levels for $x(t)$. However, we suppose that experimental protein levels, when low around a trough phase, can be susceptible to measurement errors. Such potentially inaccurate data, if these data underestimate the protein levels around the trough phase, can lead to the overestimation of $r_{\min}$ in Eq. (4) and c_{g} in Eq. (5), and thereby exaggerate a difference between c_{g} and c. To mitigate these possibly erroneous effects, we consider a new $x(t)$ whose values at $t = 0$ h, 22 h, and 24 h are replaced by that of $x(t = 2$ h), as plotted in Supplementary Fig. 3b. With this smoothened $x(t)$, we obtain $r_{\min} \approx 0.69$ h^{-1}, which is smaller than $r_{\min} \approx 0.88$ h^{-1} from the original $x(t)$. Likewise, new $c_{\mathrm{g}} \approx 0.32$ h^{-1} and $c \approx 0.12$ h^{-1}. Here, c is calculated from the newly estimated $r(t)$ in Supplementary Fig. 3c. On the other hand, without a correction for 2 h $\leq t \leq$ 10 h as in Supplementary Fig. 3d, $c \approx 0.22$ h^{-1}. Still, the cost of PRR7 waveform maintenance is at most one- to two-thirds of the assumed cost in the case of a constant degradation rate. These results are summarized in Supplementary Table 1.

Analysis of PRR5 data. In the case of the *PRR5* gene in the *Arabidopsis* circadian system, we obtained the experimental data of the mRNA and protein profiles from Fig. 5(a) of Nakamichi et al.[2] and Fig. 5(e) of Flis et al.[1], respectively. Both datasets have 2-hour sampling intervals under 12L:12D cycles. These mRNA and protein levels were normalized by the peak levels of their splines, and adopted for $g_{\mathrm{m}}(t)$ and $x(t)$ in Eq. (18), respectively. We obtained the experimental protein degradation rates by fitting exponential functions to the protein abundance data in Fig. 7(c) of Baudry et al.[4] from the wild type treated with CHX at ZT12 and then kept in the light, and in

Fig. 9(c) of Wang et al.[5] from the wild type transferred to continuous light at ZT0 and treated with CHX after 19 h. They were adopted for $r(t)$ at $t = 12$ h and 19 h ($r(t = 12\text{ h}) \approx 0.23\text{ h}^{-1}$ and $r(t = 19\text{ h}) \approx 0.41\text{ h}^{-1}$). Note that this $r(t = 19\text{ h})$ from the plants long exposed to constant light may overestimate actual $r(t = 19\text{ h})$ in 12L:12D cycles, to which all the other data pertain; however, we use this $r(t = 19\text{ h})$ because of the scarcity of available data. When calculating the cost of protein production, we use $r(t = 18\text{ h})$ that is estimated to equal $r(t = 19\text{ h})$, in order to harness the experimental protein level $x(t)$ available at $t = 18$ h. Following a similar procedure to the case with PRR7, we obtain $r_{\min} \approx 1.69\text{ h}^{-1}$ and $c_{\text{g}} \approx 0.77\text{ h}^{-1}$ (we will assume constant translation rate $k(t) \approx k$ described in Methods. In the case with time-varying $k(t)$, refer to Methods for detailed analysis).

When calculating c based on $r(t)$ in Eq. (20), we replace every $r(t) > r_{\min}$ by $r_{\min}$, because the real degradation rate is unlikely to be larger than $r_{\min} \approx 1.69\text{ h}^{-1}$. We also replace every $r(t) < \min\{r(t = 12\text{ h}), r(t = 19\text{ h})\}$ by $\min\{r(t = 12\text{ h}), r(t = 19\text{ h})\}$, and therefore the lower bound of $r(t)$ is set to the minimum value of experimental $r(t)$ values. In such a way, the difference between c and c_{g} is reduced, leading to a conservative estimate of that difference. The resulting $r(t)$ is presented in Supplementary Fig. 4a. Because $r(t)$ at 6 h $\leq t \leq$ 10 h is improbably deviated from the overall trend of experimental $r(t)$ values, we correct this part by linear extrapolation of the experimental $r(t = 12\text{ h})$ value, as shown in Fig. 3d. Consequently, $c \approx 0.17c_{\text{g}}$ with $r(t)$ in Fig. 3d and $c \approx 0.34c_{\text{g}}$ with $r(t)$ in Supplementary Fig. 4a. In other words, whether correcting $r(t)$ at 6 h $\leq t \leq$ 10 h or not, the actual cost of PRR5 waveform maintenance would be at most one-sixth to one-third of the assumed cost in the case of a constant degradation rate.

To mitigate the aforementioned, possibly erroneous effects from low protein levels around a trough phase in the case with PRR7, we consider new $x(t)$ whose values at $t = 0$ h, 22 h, and 24 h are increased as in Supplementary Fig. 4b. With this smoothened $x(t)$, we obtain $r_{\rm min} \approx 0.55$ h^{-1}, which is smaller than $r_{\rm min} \approx 1.69$ h^{-1} from the original $x(t)$. Likewise, new $c_{\rm g} \approx 0.26$ h^{-1} and $c \approx 0.13$ h^{-1}. Here, c is calculated from the newly estimated $r(t)$ in Supplementary Fig. 4c. On the other hand, without a correction for 6 h $\leq t \leq$ 10 h as in Supplementary Fig. 4d, $c \approx 0.17$ h^{-1}. Still, the cost of PRR5 waveform maintenance is at most one-half to two-thirds of the assumed cost in the case of a constant degradation rate. These results are summarized in Supplementary Table 1.

Analysis of PER2 data. In the case of the mouse PER2 protein, we obtained the time-course abundance data used in Fig. 1(a) (CHX-untreated control data) of Zhou et al.[6], and adopted this protein profile for $x(t)$. The original profile covers ∼45-hour-long data with 0.1-hour resolution. Therefore, we considered the data at 9.6 h $\leq t \leq$ 33 h for one circadian period ($T = 23.4$ h), and smoothened them with a moving window average (3-hour window). These data were normalized by their peak level, and the resulting $x(t)$ appears in Fig. 4a. $R(t)$ derived from this $x(t)$ is very noisy, and therefore smoothened with a moving window average (1-hour window). The resulting $R(t)$ gives rise to $r_{\rm min} \approx 0.47$ h^{-1}, as shown in Fig. 4b. For the experimental protein degradation rates, we used the instantaneous half-lives after 0.5 hours since CHX treatment at $t = 19$ h, 22 h, 25 h, 28 h, and 30 h in Fig. S1(a) of Zhou et al.[6]. These $r_{\rm min}$ and $r(t)$ lead to $c_{\rm g} \approx 0.23$ h^{-1} and $c \approx 0.11$ h^{-1}, as presented in Supplementary Table 1. In other words, the actual cost of PER2 waveform maintenance would be about half of the assumed cost in the case of a constant degradation rate.

Analysis of CCA1 and TOC1 in the algal clock. In the case of CCA1 and TOC1 proteins in the *Ostreococcus* circadian system, we obtained the full time-course degradation rate $r(t)$ and protein level $x(t)$ data from Fig. 1(a) and (b) of van Ooijen et al.[7], respectively (12L:12D-cycle condition). We did not perform any normalization of $x(t)$, and the unit of $x(t)$ here follows that of van Ooijen et al.[7] (molecules/cell). Because $x(t)$'s sampling resolution was rather low (4-hour sampling interval), we did not apply $r(t)$ and $x(t)$ to Eq. (3) wherein the specific form of $R(t)$ could be sensitive to the $x(t)$'s sampling resolution. For the calculation of $c_{\rm g}$, we estimated $r_{\rm min}$ as $r_{\rm min} \approx \min\{\max_t r(t), \max_t R(t)\}$, with regards to possibly-inaccurate $R(t)$ from the low sampling resolution of $x(t)$. For the calculation of c, we adopted $r(t)x(t)$ in Fig. 1(c) of van Ooijen et al.[7]. As a result, for CCA1 and TOC1, $r_{\rm min} \approx 0.25$ and 0.28 h^{-1}, $c_{\rm g} \approx 60.7$ and 19.7 molecules/cell/h, and $c \approx 42.5$ and 11.6 molecules/cell/h, respectively. In other words, the cost of CCA1 and TOC1 production is about two-thirds of the assumed cost in the case of constant degradation rates.

Description of the fungal clock. Equation (8) or (9) does not only describe mammalian and insect clocks, but also the fungal clock. In the core clock of the fungus *Neurospora crassa*, WHITE COLLAR-1, 2 (WC-1 and WC-2) proteins form a WHITE COLLAR COMPLEX (WCC) that activates the expression of *frequency* (*frq*) gene. The expressed FRQ protein subsequently blocks the WCC activity in the nucleus by the clearance of WC-1[8] or the inactivation of WCC[9], forming a negative feedback loop. Accordingly, nuclear WC-1 and FRQ concentrations can be mapped to $x_{\rm A}(t)$ and $y(t)$ in Eq. (8) (or, $x(t)$ and $y(t)$ in Eq. (9)), respectively[10]. Because *wc-1* mRNA levels are almost constant over time[11], $g_{\rm A}(t)$ in the fungal case would be almost constant.

Phase difference between animal clock components with small τ in Eq. (14). If $\tau \ll T$ in Eq. (14), Eq. (17) can be used to calculate a phase difference between $x(t)$ and $y(t)$. Without loss of generality, let $x(t)$ be the lowest at $t = T$, i.e., $t_{\frac{1}{x}} = T$. Depending on signs of α and $1 - \beta\tau$ in Eq. (17), we consider the following four cases:

1. If $\alpha > 0$ and $\beta\tau < 1$, $y(t)$ in Eq. (17) is described essentially in the same way as Eq. (10), while extra constants in Eq. (17) do not affect the way to determine a phase difference between $x(t)$ and $y(t)$. Therefore, t_y still follows

   $$t_{-\frac{x'}{x}} \leq t_y \leq t_{\frac{1}{x}} = T,$$

   and the phase difference between $x(t)$ and $y(t)$ is determined in a similar way to the case with constant $g_{\mathrm{A}}(t)$ (i.e., $g_{\mathrm{A}}(t) = g$). $y(t)$ in this case will be called $y_1(t)$.

2. If $\alpha > 0$ and $\beta\tau > 1$, $y(t)$ is determined in a similar way to $y_1(t)$, but with the flipped sign of $x'(t)$. Therefore,

   $$0 \leq t_y \leq t_{\frac{x'}{x}}.$$

   $y(t)$ in this case will be called $y_2(t)$.

3. If $\alpha < 0$ and $\beta\tau < 1$, $y(t)$ is described in a similar way to $-y_2(t)$. Therefore,

   $$t_x \leq t_y \leq t_{-\frac{x'}{x}}.$$

4. If $\alpha < 0$ and $\beta\tau > 1$, $y(t)$ is described in a similar way to $-y_1(t)$. Therefore,

   $$t_{\frac{x'}{x}} \leq t_y \leq t_x.$$

Analysis of Eq. (14) with large τ. In addition to the case of Eq. (14) with $\tau \ll T$, we analyze the case with $\tau \sim T/2$. In this case, $\tau = T/2 + \epsilon$ with $|\epsilon| \ll T$, and $x(t+\tau)$ in Eq. (14) can be approximated as

$$x\left(t + \frac{T}{2} + \epsilon\right) \approx x\left(t + \frac{T}{2}\right) + \epsilon x'\left(t + \frac{T}{2}\right).$$

We further assume the waveform that $x(t + T/2) \approx J - x(t)$, where J is a constant satisfying $J \approx (2/T)\int_0^T x(t)dt$. From Eq. (9),

$$ky(t) \approx \frac{\alpha + \beta J - (1+\beta\epsilon)x'(t)}{x(t)} - (r_0 + \beta).$$

This equation takes a similar form to Eq. (17). By dividing four different categories of $y(t)$ depending on signs of $\alpha + \beta J$ and $1 + \beta\epsilon$, it is straightforward to obtain similar results to our previous analysis of a phase difference between $x(t)$ and $y(t)$ when $\tau \ll T$.

In addition, both $x(t)$ and $y(t)$ can have symmetric waveforms as long as $|(\alpha + \beta J)/(1 + \beta\epsilon)| \gg \max_t |x'(t)|$ (for example, this condition can be satisfied when $\beta\epsilon \approx -1$).

Phase difference between $x(t)$ and $y(t)$ in the case of Eqs. (13) and (14). To illustrate the diverse phase differences conferred by BMAL1 cycling, we study the case with a sinusoidal wave $x(t)$ in Eq. (13) and consider the oscillation of $g_{\mathrm{A}}(t)$ in Eq. (14). From Eq. (9), t_y is obtained as

$$\begin{aligned} \omega t_y &= 2\pi n + 2\tan^{-1}\left\{ \frac{\omega\alpha + C\beta[1-\cos(\omega\tau)]}{(C+L)[\omega - \beta\sin(\omega\tau)]} \right. \\ &\quad \left. - \frac{\sqrt{(\omega\alpha + C\beta[1-\cos(\omega\tau)])^2 + (C^2 - L^2)[\omega - \beta\sin(\omega\tau)]^2}}{(C+L)[\omega - \beta\sin(\omega\tau)]} \right\} \end{aligned} \quad (1)$$

with $C = h_0\omega + L$ and an integer n that satisfies $0 < \omega t_y \leq 2\pi$. From Eq. (15), α satisfies

$$\alpha \geq \alpha_{\min} = \max\left\{ L\sqrt{1 - \frac{2\beta}{\omega}\sin(\omega\tau) + \frac{\beta^2}{\omega^2}} - \frac{\beta L}{\omega}, 0 \right\} - \beta h_0. \quad (2)$$

Equation (1) and $t_x = T/2$ give rise to the exact solution of the phase difference ϕ between $x(t)$ and $y(t)$ ($\phi = |\omega(t_x - t_y)|$), as plotted in Fig. 5e. This exact solution is in good agreement with our generic results based on the approximation Eqs. (16) and (17).

Supplementary References

1. Flis, A. *et al.* Defining the robust behaviour of the plant clock gene circuit with absolute RNA timeseries and open infrastructure. *Open Biology* **5**, 150042 (2015). URL `http://dx.doi.org/10.1098/rsob.150042`.

2. Nakamichi, N. *et al.* PSEUDO-RESPONSE REGULATORS 9, 7, and 5 are transcriptional repressors in the *Arabidopsis* circadian clock. *The Plant Cell* **22**, 594–605 (2010). URL `http://dx.doi.org/10.1105/tpc.109.072892`.

3. Farré, E. M. & Kay, S. A. PRR7 protein levels are regulated by light and the circadian clock in *Arabidopsis*. *The Plant Journal* **52**, 548–560 (2007). URL `http://dx.doi.org/10.1111/j.1365-313x.2007.03258.x`.

4. Baudry, A. *et al.* F-Box proteins FKF1 and LKP2 act in concert with ZEITLUPE to control *Arabidopsis* clock progression. *The Plant Cell* **22**, 606–622 (2010). URL `http://dx.doi.org/10.1105/tpc.109.072843`.

5. Wang, L., Fujiwara, S. & Somers, D. E. PRR5 regulates phosphorylation, nuclear import and subnuclear localization of TOC1 in the *Arabidopsis* circadian clock. *The EMBO Journal* **29**, 1903–1915 (2010). URL `http://dx.doi.org/10.1038/emboj.2010.76`.

6. Zhou, M., Kim, J. K., Eng, G. W., Forger, D. B. & Virshup, D. M. A Period2 phosphoswitch regulates and temperature compensates circadian period. *Molecular Cell* **60**, 77–88 (2015). URL `http://dx.doi.org/10.1016/j.molcel.2015.08.022`.

7. van Ooijen, G., Dixon, L. E., Troein, C. & Millar, A. J. Proteasome function is required for biological timing throughout the twenty-four hour cycle. *Current Biology* **21**, 869–875 (2011). URL `http://dx.doi.org/10.1016/j.cub.2011.03.060`.

8. Dunlap, J. C. & Loros, J. J. How fungi keep time: circadian system in *Neurospora* and other fungi. *Current Opinion in Microbiology* **9**, 579–587 (2006). URL `http://dx.doi.org/10.1016/j.mib.2006.10.008`.

9. Schafmeier, T. *et al.* Transcriptional feedback of *Neurospora* circadian clock gene by phosphorylation-dependent inactivation of its transcription factor. *Cell* **122**, 235–246 (2005). URL `http://dx.doi.org/10.1016/j.cell.2005.05.032`.

10. Hong, C. I., Jolma, I. W., Loros, J. J., Dunlap, J. C. & Ruoff, P. Simulating dark expressions and interactions of *frq* and *wc-1* in the *Neurospora* circadian clock. *Biophysical Journal* **94**, 1221–1232 (2008). URL `http://dx.doi.org/10.1529/biophysj.107.115154`.

11. Merrow, M. *et al.* Circadian regulation of the light input pathway in *Neurospora crassa*. *The EMBO Journal* **20**, 307–315 (2001). URL `http://dx.doi.org/10.1093/emboj/20.3.307`.

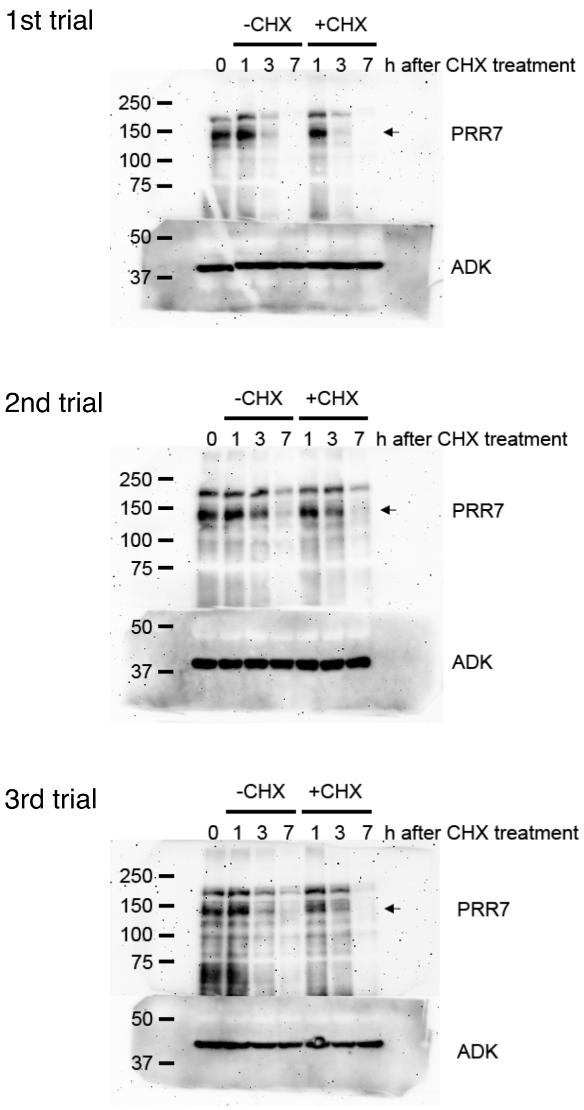

Supplementary Figure 1: Representative immunoblots of PRR7 proteins following cycloheximide (CHX) treatment at ZT17. After addition of CHX or mock (ethanol) to the 14-day-old plants grown in 12L:12D, FLAG-PRR7-GFP levels were analyzed by anti-GFP antibodies. The bands corresponding to PRR7 proteins are indicated by an arrow. Adenosine kinase (ADK) was used as a loading control.

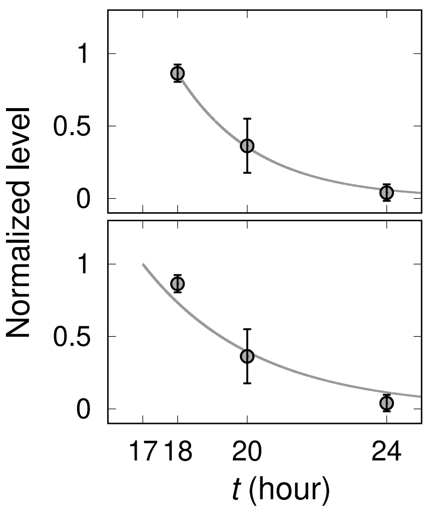

Supplementary Figure 2: Our experimental measurement of PRR7 levels after CHX treatment at ZT17. PRR7 levels are normalized to the levels at ZT17. Circles and error bars represent the averages and standard deviations from three biological repeats, respectively. Considering a lag time for the full effect of CHX, an exponential function is fitted to the data from ZT18 (gray solid line in the upper panel), leading to the degradation rate $r(t = 18$ h$) \approx 0.45$ h^{-1}. Alternatively, an exponential function can be fitted to the data from ZT17 (gray solid line in the lower panel), leading to $r(t = 17$ h$) \approx 0.31$ h^{-1}.

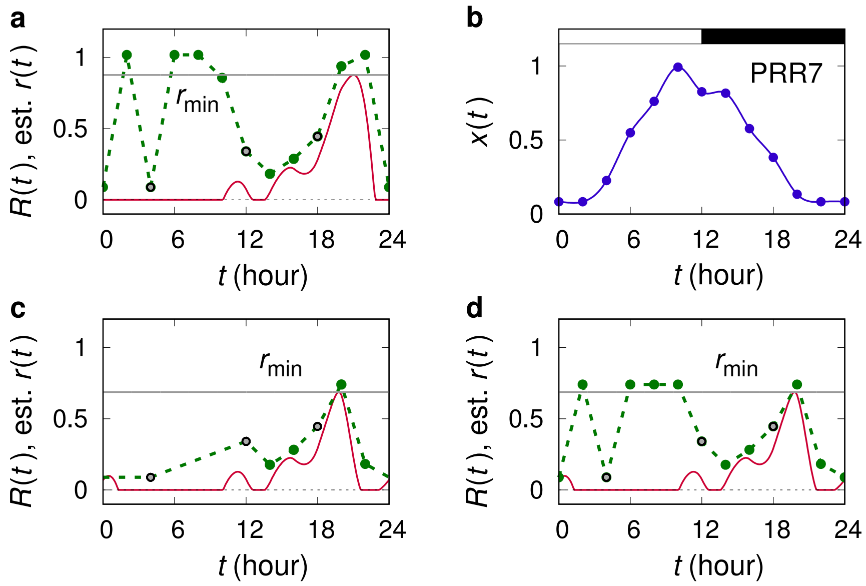

Supplementary Figure 3: Estimated $r(t)$ curves of PRR7 proteins under the assumption of constant k. For the definition of each notation, refer to Eqs. (1), (3), (4), and (18). (**a**) Estimated $r(t)$ without a correction for 2 h $\leq t \leq 10$ h (green circles and dashed line) is plotted with $R(t)$ (red solid line) and $r_{\min}$ (gray solid line). These quantities are derived from $x(t)$ and $g_{\mathrm{m}}(t)$ in Fig. 2 and experimental degradation rates (gray-filled black circles)[3]. The degradation rate at $t = 18$ h comes from our own experimental data in Supplementary Fig. 2. (**b**) The same $x(t)$ as Fig. 2a but with modified protein levels around a trough phase. (**c**) and (**d**) The modified protein profile in **b** is used for $R(t)$ (red solid line), $r_{\min}$ (gray solid line), and estimated $r(t)$ (green circles and dashed line). This estimated $r(t)$ with and without a correction for 2 h $\leq t \leq 10$ h is presented in **c** and **d**, respectively, along with experimental degradation rates (gray-filled black circles). In **a**, **c**, and **d**, the vertical axis unit is h^{-1}. In **b**, white and black segments correspond to light and dark intervals, respectively.

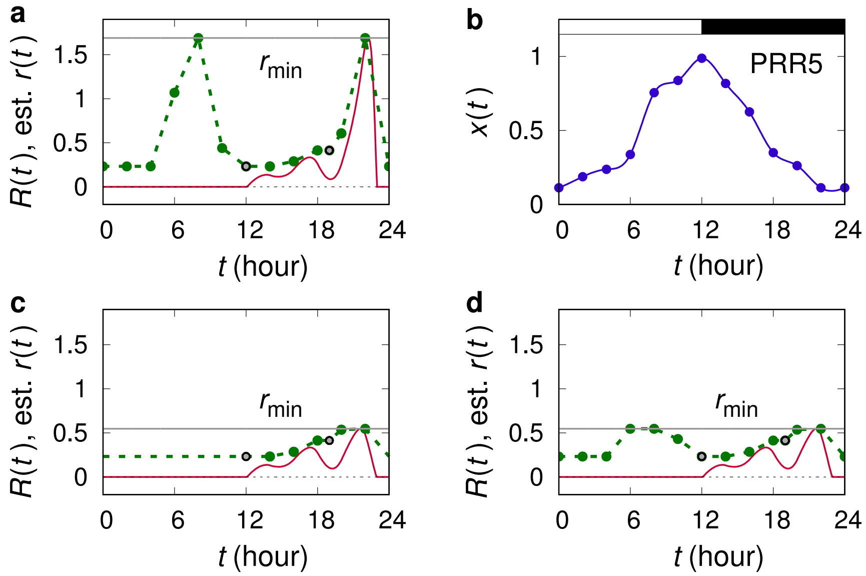

Supplementary Figure 4: Estimated $r(t)$ curves of PRR5 proteins under the assumption of constant k. For the definition of each notation, refer to Eqs. (1), (3), (4), and (18). (**a**) Estimated $r(t)$ without a correction for 6 h $\leq t \leq 10$ h (green circles and dashed line) is plotted with $R(t)$ (red solid line) and $r_{\min}$ (gray solid line). These quantities are derived from $x(t)$ and $g_{\mathrm{m}}(t)$ in Fig. 3 and experimental degradation rates (gray-filled black circles)[4,5]. (**b**) The same $x(t)$ as Fig. 3a but with modified protein levels around a trough phase. (**c**) and (**d**) The modified protein profile in **b** is used for $R(t)$ (red solid line), $r_{\min}$ (gray solid line), and estimated $r(t)$ (green circles and dashed line). This estimated $r(t)$ with and without a correction for 6 h $\leq t \leq 10$ h is presented in **c** and **d**, respectively, along with experimental degradation rates (gray-filled black circles). In **a**, **c**, and **d**, the vertical axis unit is h^{-1}. In **b**, white and black segments correspond to light and dark intervals, respectively.

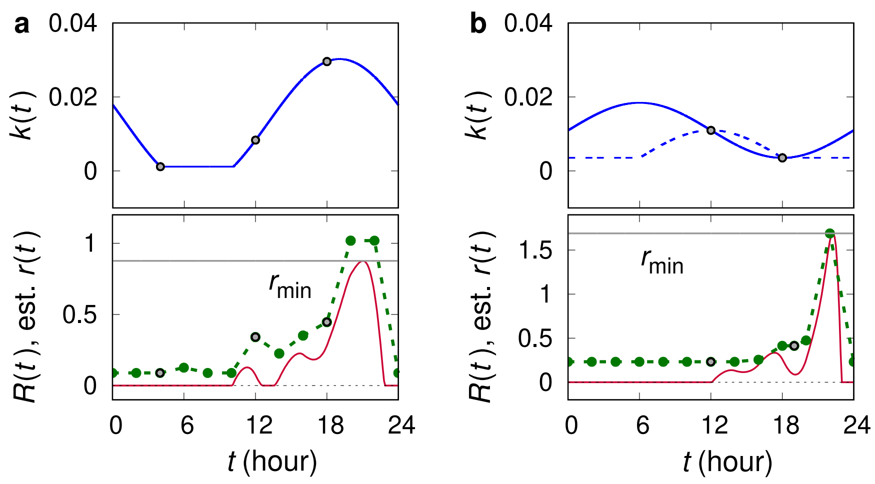

Supplementary Figure 5: Estimated $r(t)$ curves of PRR7 and PRR5 proteins under the assumption of sinusoidally time-varying $k(t)$. For the definition of each notation, refer to Eqs. (1), (3), (4), (18), (19), and Methods. (**a**) PRR7 and (**b**) PRR5. In each upper panel, circles and lines denote the right-hand side values of Eq. (19) and the fitted $k(t)$, respectively. The upper panel in **b** shows the $k(t)$s with $\phi = 0$ (solid line) and $\phi = \pi/2$ (dashed line). In each lower panel, estimated $r(t)$ (green circles and dashed line) is plotted with $R(t)$ (red solid line), $r_{\min}$ (gray solid line), and experimental degradation rates (gray-filled black circles). This $r(t)$ was estimated in a similar way to Figs. 2f and 3d. The lower panel in **b** demonstrates the case of $\phi = 0$ in $k(t)$, and the other ϕ values (such as $\phi = \pi/2$ in the upper panel) do not show much different results. The vertical axis unit is h^{-1} for all upper and lower panels.

Supplementary Table 1: Estimated values of $r_{\rm min}$, $c_{\rm g} = r_{\rm min}\langle x(t)\rangle$, and $c = \langle r(t)x(t)\rangle$ as well as cost reduction for PRR7, PRR5, and PER2. For the definitions of $c_{\rm g}$ and c, refer to Eqs. (2) and (5). The cost reduction due to the time- or phase-specific $r(t)$ is defined as $(c_{\rm g} - c)/c_{\rm g}$. We here assume constant k in Eq. (18). We treat $x(t)$ as dimensionless through the normalization of $x(t)$ by its peak value (Figs. 2a, 3a, and 4a), and thus units of $r_{\rm min}$, $c_{\rm g}$, and c in the Table are hour^{-1}. The cost reduction itself is not a quantity affected by the normalization of $x(t)$, and hence there is no loss of generality in its values. †The result from smoothened $x(t)$ of each protein around a trough phase. ‡The result without a correction for 2 h $\leq t \leq 10$ h (PRR7) or for 6 h $\leq t \leq 10$ h (PRR5).

Protein	$r_{\rm min}$ (h^{-1})	$c_{\rm g}$ (h^{-1})	c (h^{-1})	Cost reduction
PRR7	0.88	0.40	0.12	∼70%
PRR7†	0.69	0.32	0.12	∼63%
PRR7‡	0.88	0.40	0.27	∼33%
PRR7†‡	0.69	0.32	0.22	∼31%
PRR5	1.69	0.77	0.13	∼83%
PRR5†	0.55	0.26	0.13	∼50%
PRR5‡	1.69	0.77	0.26	∼66%
PRR5†‡	0.55	0.26	0.17	∼35%
PER2	0.47	0.23	0.11	∼52%
