# Supplementary material for: Waveforms of molecular oscillations reveal circadian timekeeping mechanisms
Source: Commun Biol. 2018 Nov 26;1:207. doi: 10.1038/s42003-018-0217-1 (PMC6255756; doi:10.1038/s42003-018-0217-1)
Supplement: Supplementary file 2 — Description of Additional Supplementary Files [file 42003_2018_217_MOESM2_ESM.docx]

**Description of Additional Supplementary Files**

**File Name**: Supplementary Data 1

**Description**: “Source data used for plotting the main figures”

Fig. 1b. These data points are schematic waveforms of clock protein profiles. They are intended to help the readers understand the meaning of the protein degradation rate described in Eq. (4).

Fig. 2–4. These data include clock protein and mRNA profiles and (experimental and inferred) protein degradation rates. The proteins are plant PRR7 (Fig. 2) and PRR5 (Fig. 3), and mammalian PER2 (Fig. 4) proteins. The protein and mRNA profiles were collected from the literature cited in the main text (Nakamichi et al.26, Flis et al.27, and Zhou et al.35). The PRR7 degradation rate at t = 18 h was obtained from our own seedling experiment described in Methods, and the rest of the experimental protein degradation rates were collected from the literature cited in the main text (Farre & Kay28, Baudry et al.29, Wang et al.30, and Zhou et al.35). The PRR7 and PRR5’s inferred protein degradation rates were obtained from Eqs. (18)–(20) by incorporating the protein and mRNA profiles and experimental protein degradation rates. The PER2’s inferred protein degradation rates were obtained from Eq. (3) by incorporating the protein profile and experimental protein degradation rates. In this manuscript, these data were used for validating the relationships between the protein waveforms and experimental protein degradation rates in Eq. (3), as well as for calculating the protein production cost reduction with time-dependent degradation rates, as presented in Table 1.

Fig. 5b–e. Phase differences between active CLOCK-BMAL1, and PER-CRY that is not binding to CLOCK-BMAL1. These figures were generated by Eqs. (9)–(14) with the sinusoidal waveform of the active CLOCK-BMAL1 profile, as fully detailed in the main text. In this manuscript, these figures were used for demonstrating that oscillating BMAL1 levels result in more diverse phase differences than constant BMAL1 levels, in the case of the mammalian clock.

**File Name**: Supplementary Software 1

**Description**: Codes used for analyzing the data in the paper.
